# IMAC capture of recombinant protein from unclarified mammalian cell feed streams

**DOI:** 10.1002/bit.25705

**Published:** 2015-09-03

**Authors:** Alexander Kinna, Berend Tolner, Enrique Miranda Rota, Nigel Titchener‐Hooker, Darren Nesbeth, Kerry Chester

**Affiliations:** ^1^ Department of Oncology, University College London, UCL Cancer Institute72 Huntley StreetLondonWC1E 6BTUK; ^2^ Department of Biochemical Engineering, University College LondonGower StreetLondonWC1E 6BTUK

**Keywords:** radial flow chromatography, downstream processing, IMAC, recombinant protein, integrated processing, Chinese hamster ovary cells

## Abstract

Fusion‐tag affinity chromatography is a key technique in recombinant protein purification. Current methods for protein recovery from mammalian cells are hampered by the need for feed stream clarification. We have developed a method for direct capture using immobilized metal affinity chromatography (IMAC) of hexahistidine (His6) tagged proteins from unclarified mammalian cell feed streams. The process employs radial flow chromatography with 300–500 μm diameter agarose resin beads that allow free passage of cells but capture His‐tagged proteins from the feed stream; circumventing expensive and cumbersome centrifugation and/or filtration steps. The method is exemplified by Chinese Hamster Ovary (CHO) cell expression and subsequent recovery of recombinant His‐tagged carcinoembryonic antigen (CEA); a heavily glycosylated and clinically relevant protein. Despite operating at a high NaCl concentration necessary for IMAC binding, cells remained over 96% viable after passage through the column with host cell proteases and DNA detected at ∼8 U/mL and 2 ng/μL in column flow‐through, respectively. Recovery of His‐tagged CEA from unclarified feed yielded 71% product recovery. This work provides a basis for direct primary capture of fully glycosylated recombinant proteins from unclarified mammalian cell feed streams. Biotechnol. Bioeng. 2016;113: 130–140. © 2015 Wiley Periodicals, Inc.

## Introduction

Expression of recombinant proteins in yeasts, bacteria, and mammalian cells is now routine practice at industrial scale. Although microbial expression systems are used to produce the majority of industrial recombinant proteins, those requiring glycosylation for correct structure and function are preferentially expressed in mammalian host systems (Dean and Reddy, 2013; Lai et al., 2013; Wurm, 2004; Zhu, 2011). The glycosylation profile of proteins from microbial cells differs from that of mammalian cells (Demain and Vaishnav, 2009). Incorrectly glycosylated proteins from microbial sources can be non‐functional (Buck et al., 2013) and even induce patient immune responses (Jacobs et al., 2008; Jefferis, 2012).

A disadvantage of mammalian cell expression is the high cost of goods/gram of product (COG/g) when compared to microbial expression. However, in the past a substantial percentage of these costs derived from fermentation, whereas recently production costs have become more heavily weighted toward downstream processing (González et al., 2003). This is largely due to improvements in specific productivity, better feeding strategies, and protein engineering; combining to produce cell densities in excess of 1 × 10^7^ cells/mL and yields of 8 g/L (Farid, 2007; Hacker et al., 2009). These advances have in turn created new challenges for downstream processing, leading to a focus on developing high capacity methods for the primary capture of protein from high cell density feeds.

**Figure 1:**
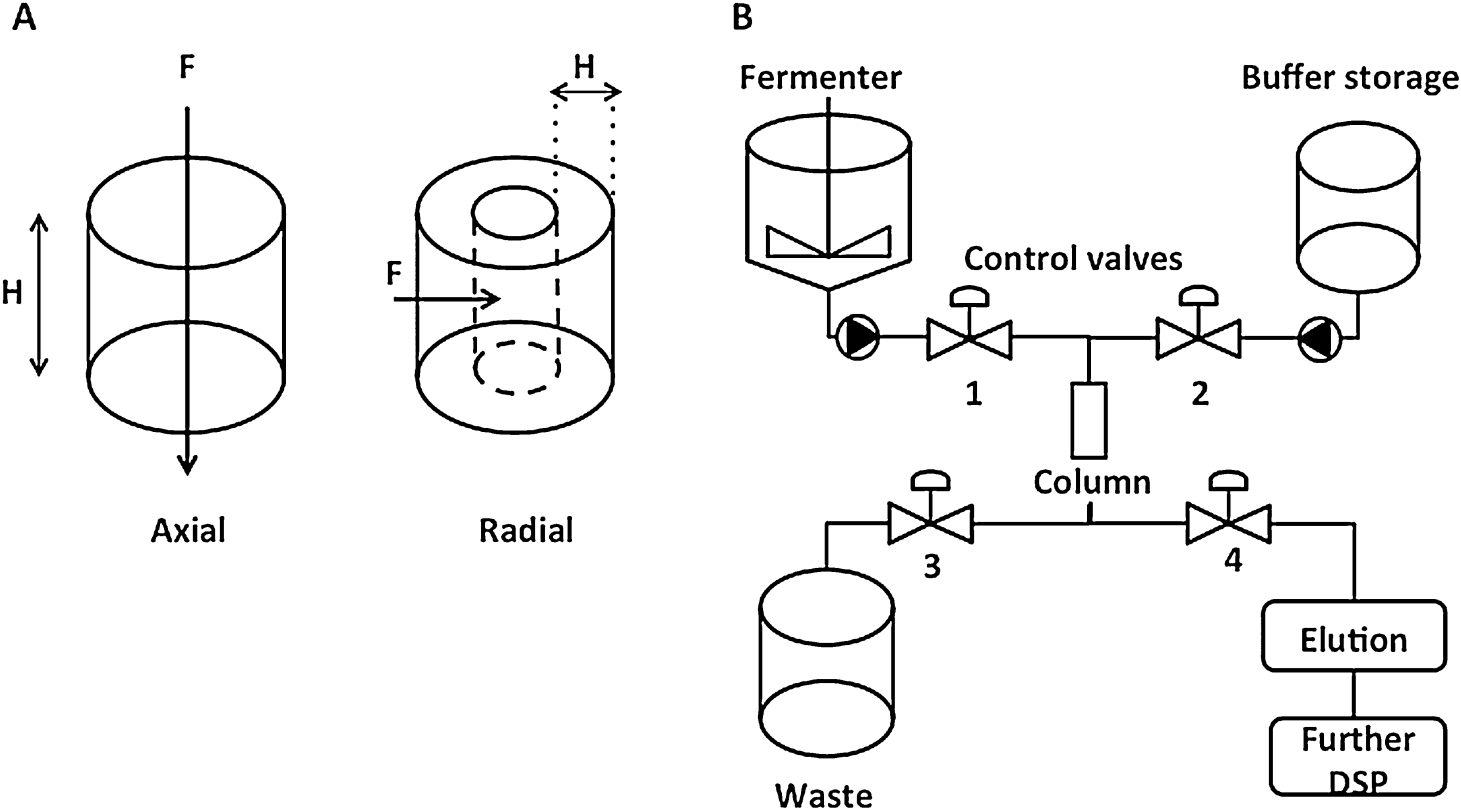
Radial flow chromatography methods. **A**: Axial and radial flow chromatography. Frits are located at the outer and inner circumferences of the radial column. Bed height (H), flow direction (F). **B**: Process flow diagram of integrated radial flow chromatography control.

Current methods for primary capture of recombinant protein necessitate solid–liquid separation to remove cells and debris, followed by chromatographic capture steps based on ion exchange or affinity. Typically, solid–liquid separation consists of centrifugation and filtration. These techniques can account for up to 25% COG/g when factors such as capital cost and energy consumption are included (Marichal‐Gallardo and Álvarez, 2012). Primary processing of high cell density feed streams requires particular consideration to avoid cell stresses and shearing that could result in protein aggregation and cell lysis (Hutchinson et al., 2006; Kiese et al., 2008). The latter affects the quantity and quality of product through the release of host cell proteins (HCP), proteases, and DNA (Sandberg et al., 2006). Attractive options for primary recovery include use of resins in the form of large diameter beads that create a highly porous bed enabling simultaneous protein capture and concentration whilst allowing cells to pass through. Such large bead diameter chromatography resins are made from agarose beads typically between 300 μm and 500 μm in diameter that can be combined with radial flow chromatography (RFC). By contrast to axial flow, RFC consists of two concentric cylindrical porous frits holding a stationary phase between them. In RFC, feed flows from the outer to the inner surface across the radius of the column (Fig. 1A) providing a minimal pressure drop. Improved elution peak resolution due to the trapezoidal column geometry and greater operational flow rates are reported as advantages (Besselink et al., 2013). RFC columns can be packed with many resins depending on frit design, including ion exchange (IEX), immobilized metal affinity chromatography (IMAC) or protein A/G and are easily scalable.

Recombinant protein purification has long relied on the use of engineered affinity tags, which can be used to facilitate capture, in addition to improving expression and solubility of proteins (Young, 2012). The polyhistidine repeat tag, however, remains the major workhorse for affinity purification due to the simplicity of amino or carboxy terminal fusion to proteins that lack a natural purification tag (Terpe, 2003). Concerns have been raised about the potential of therapeutically redundant purification tags to affect in vivo half life, therapuetic function of proteins or be implicated in immunogenicity. Although tags can be removed after purification (Waugh, 2011), His‐tagged proteins have been used in a number of clinical products without adverse effects (Tolner et al., 2013, Table 37.1). Data present in GenBank (http://www.ncbi.nlm.nih.gov/genbank) also reveal a variety of human proteins containing stretches of six or more consecutive histidines. The occurrence of these sequences in human proteins makes this sequence an unlikely candidate to be involved in immunogenicity.

IMAC offers an inexpensive, robust, and versatile approach to protein capture chromatography (Shukla et al., 2001). In addition to the recovery of tagged proteins, IMAC capture of recombinant proteins with naturally occurring histidine rich regions has also been demonstrated, including antibodies and antibody fragments (Cheung et al., 2012; Todorova‐Balvay et al., 2004). Hexahistidine (His) tagged proteins have been IMAC captured without the need for feed stream clarification from microbial hosts previously (Tolner, 2006; Willoughby, 2004), however, direct capture from mammalian cells is more challenging due to the increased sensitivity of cells to shear and also the high salt concentration required for IMAC binding.

Here, we report the development of a process using RFC packed with a large diameter resin to obtain an integrated primary capture step for unclarified CHO cell fermentation feed streams. The method was developed using suspension‐adapted CHO cells grown in shake flask culture and purified on a micro‐RFC column with 6 cm bed height loaded with Sterogene CellThru Bigbead IMAC resin. The process was then scaled up to a 1 L feed whilst chromatography bed height was maintained. We based the study upon recovery of CEA, a clinically relevant glycoprotein used in many diagnostic and therapeutic applications (Francis et al., 2002; Girgis et al., 2011; Graff et al., 2004; Kaushal et al., 2008; Prager et al., 2014). CEA has previously proved difficult to manufacture as a complete glycoprotein in recombinant form and attempts to express it in yeasts have yielded only truncated forms (Hellwig, 1999; Sainz‐Pastor, 2006; Tolner, 2013), hence the demand has been met by recovery from ascetic fluids (Hammarström, 1999; Sigma–Aldrich, St. Louis, MO). CEA is 50–60% glycosylated by weight making microbial expression unsuitable (Paxton, 1987). The study forms the basis of a primary capture step applicable to the recovery of many recombinant proteins from unclarified mammalian cell feed streams.

## Materials and Methods

### Cell Lines and Maintenance

Suspension and serum free adapted Chinese Hamster Ovary cells (CHO‐S) (Life Technologies, Paisley, UK) or CHO‐S expressing His‐tagged CEA (CHO‐CEA) were seeded at a density of 0.3 million viable cells (MVC)/mL in 300 mL of a 1:1 (v/v) mix of CD‐CHO (Life Technologies) and EX‐CELL CD‐CHO 3 (Sigma–Aldrich) in 1 L Erlenmeyer flasks (Corning Limited, Union City, CA) with 0.22 μm vented caps to a maximum cell density of 3 × 10^6^ cells/mL. All incubation conditions were 125 rpm, 8% CO_2_, and 37°C.

### Cell Counting, Viability, and Size Distribution

A Vi‐CELL XR automated cell counter (Beckman Coulter, High Wycombe, UK) was used to determine cell density and viability via the Trypan blue exclusion method. Vi‐CELL parameters were min/max size 6–50 μm and 50 counts. Particle size distribution was analyzed using the CASY Cell Counter Analyzer System (Roche, Basal, Sweden) with analyses parameters set to min/max 3–40 μm. Cell culture was diluted in phosphate buffered saline (PBS) where appropriate to ensure accuracy in cell counting and particle distribution analyses.

### Media Bioprofiling

Media composition and cell culture metabolites were analyzed by ion selective electrode potentiometry, amperometry, and enzymatic reaction‐dependent biosensors in 1 mL aliquots immediately after sample removal (BioProfile 400, Nova Biomedical, Inc., Waltham, MA).

### Radial Flow Chromatography Operation

A scale down RFC column with bed volume of 5 mL and height of 6 cm was used with either 40 μm or 200 μm pore size frits (Proxcys, Nieuw‐Amsterdam, Netherlands) (Fig. 1A). The column was packed with iminodiacetic acid (IDA) Chelating Cellthru^TM^ BigBead agarose resin (Sterogene, Carlsbad, CA) as per the manufacturers instructions. Column conditioning was performed to remove air bubbles with 6× column volumes (CV) PBS (pH 7.2). The resin was charged with 5CV 100 mM nickel sulphate and equilibrated with 0.5 M NaCl/ 0.5× PBS/ 10 mM imidazole. An operational flow rate of 1 CV/min was used for column loading, achieved using a peristaltic pump (New MBR, Zurich, Switzerland). Whilst frit pore sizes of both 200 μm and 40 μm were studied, the 200 μm pore frit was used routinely. Non‐specifically bound proteins were removed via wash buffer (40 mM imidazole/ 0.5X PBS/ 0.5 M NaCl) before elution with 200 mM imidazole/0.5X PBS/ 0.5 M NaCl. The column was stripped and regenerated after each use using 50 mM NA‐EDTA and stored in 20% (v/v) ethanol.

### Feed Clarification

Where feed was clarified, centrifugation at 500*g* for 20 min in an Avanti J‐26 XPI (Beckman Coulter, Woerdan, The Netherlands) was used to remove solids before filtration through a 500 mL 0.22 μm Stericup filter unit (Corning Limited).

### Bioreactor Fermentation

A single‐use pneumatic bioreactor from PBS Biotech, Inc (CA) with a working volume of 3 L was used for scaled up cell culture. Sterile polycarbonate gamma‐irradiated SUT 3 L units (PBS Biotech Inc, CA) were seeded with CHO‐CEA cells at a density of 0.3 × 10^6^ cells/mL. Reactor conditions were set at 37°C with 25 RPM pneumatic vertical wheel agitation, pH 7.2 and >30% DO_2_ maintained via PID control (Kim et al., 2013). Cells were maintained in a fed‐batch mode of operation with 3% (v/v) EfficientFeed B (Life Technologies) daily from Day 4. Once cells reached the end of the exponential growth phase temperature and agitation were reduced to 35°C and 20 RPM, respectively (Nam et al., 2008).

### Column‐Reactor Integration and Harvest

In‐line integration of RFC was achieved via welding the column tubing directly to the reactor harvest line using Masterflex C‐FLEX tubing (Cole‐Palmer, London, UK) with a sterile tube welder (GE Healthcare, Buckinghamshire, UK). Valve and pump operation was used to control flow from the reactor (Valves 1 and 3 open), followed by wash, elution and regeneration buffers (Valves 2 and either 3 or 4 open) (Fig. 1B). Cells were buffered to IMAC binding conditions by addition of 4x concentrated IMAC buffer to the reactor prior to passage directly through the column in 3 × 1 L batches. The column was eluted and regenerated between batches as described above.

### Protease Activity Quantification

Protease activity in the supernatant and column flow‐through was determined using the fluorescence resonance energy transfer (FRET) protease assay (Life Technologies) according to the manufactures instructions. Briefly, 100 mL of casein labeled stock solution (10 mg/mL) or assay buffer as blank was added to each well of a black 96‐well plate. Supernatant (50 mL) was added and incubated for 20–30 min at room temperature before addition of 50 mL TNBSA solution and a further incubation of 20 min at room temperature. The assay plate was read in a Varioskan Flash plate reader (Life Technologies) in fluorescence mode at 485/538 nm. Activity was quantified against a standard curve of trypsin with known proteolytic activity, 15,525 U/mg (1 U defined as a change in absorbance at 253 nm of 0.001/min at 25°C, pH 7.6 in a reaction volume of 3.2 mL of Na‐Benzoyl‐l‐Arginine Ethyl Ester Solution (BAEE) (Papaioannou and Liener, 1968).

### Real‐Time qPCR

Real time PCR (adapted from Nissom, 2007) was performed using a Realplex4 Mastercycler EPgradient S (Eppendorf, Stevenage, UK) using the following conditions: initial heat denaturation at 50°C for 2 min, 95°C for 10 min, followed by 40 cycles each of 95°C for 15 s and 60°C for 1 min. Primers directed against genomic DNA isolated from *Critcetulus griseus* (CHO) were synthesized by IDT (Integrated DNA Technologies, Glasgow, UK). Primer sequences were: Sense 5′ACAGGTTTCTGCTTCTGGCT and Anti‐sense 5′CATCAGCTGACTGGTTCACA. The reaction mix contained 3 μL of sample in a 25 μL reaction mixture of ITAQ SYBR with a final concentration of 5 nM of each primer.

### Enzyme Linked Immunosorbant Assay (ELISA)

CEA was quantified using a double antibody sandwich ELISA method. A 96‐well plate (Life Technologies) was coated with 10 μg/mL polyclonal rabbit anti‐CEA (UCL Cancer Institute, London). After washing with PBS twice, wells were blocked with 200 μL of 5% (w/v) skimmed milk in PBS. Plates were washed as before and samples were added along with a serial dilution of His‐tagged CEA (R&D, Abingdon, UK) to form a standard curve. After washing, 1 μg/mL murine anti‐CEA was added for 1 h and detected using anti‐mouse‐horseradish peroxidase (HRP) conjugate (R&D, Abingdon, UK) diluted 1:4,000 in phosphate buffered saline (PBS), using 0.5 mg/mL o‐phenylenediamine dihydrochloride (Sigma–Aldrich) in phosphocitrate buffer for development. Color was analyzed by absorbance at 490 nm in a Varioskan Flash platereader (Life Technologies). All incubations were carried out at room temperature.

### Model His‐Tagged Protein Recovery

A His‐tagged ScFv (SM3E) (Graff et al., 2004) was expressed and purified from *Pichia pastoris* as previously described (Tolner et al., 2006) and used as the test protein for recovery from cell culture media. Ultraviolet light absorbance and conductivity were analyzed by linking the RFC to an Akta Prime Plus (GE Healthcare). Eluted fractions were resolved by 10% (v/v) sodium dodecyl sulphate–polyacrylamide gel electrophoresis (SDS–PAGE), stained for 1 h with Coomassie Brilliant Blue (Sigma–Aldrich) and destained until clear using a mixture of 10% (v/v) glacial acetic acid, 30% (v/v) methanol, and 60% (v/v) double distilled water (ddH_2_O).

### Western Blotting

Purified His‐tagged CEA was quantified via ELISA assay and diluted to 1 μg/mL before being resolved by SDS–PAGE as above and transferred to polyvinylidene difluoride (PVDF) membranes (BioRad, Hercules, CA). His‐tagged CEA (R&D) was used as positive control. Membranes were blocked with 5% (w/v) skimmed milk powder in 0.1% (v/v) PBS and probed with murine anti‐His tag (Qiagen, Limburg, Netherlands) or an anti‐CEA antibody (Pedley et al., 1987). Bound antibodies were detected with HRP‐linked secondary anti‐mouse‐HRP conjugated antibody (R&D, Abingdon, UK) and developed using Luminata Classico Western HRP substrate (Millipore, Darmstadt, Germany).

### Design of Experiments

A central composite design (CCD) was used for optimization of buffer conditions for His‐tagged CEA Ni‐IMAC binding. Three variables were investigated with parameters based on standard IMAC binding buffers; (A) pH, (B) NaCl concentration (mM), and (C) imidazole concentration (mM). Clarified supernatant (100 mL) containing His‐tagged CEA was adjusted to the appropriate buffer composition and randomized using the design matrix (Table I) prior to capture using a 5 mL HisTrap column (GE Healthcare). CEA yield was assessed via ELISA and process interactions were analyzed using Design‐Expert 8 (Stat‐Ease, Inc., MN).

**Table I:** Design Matrix for Optimization of IMAC Binding Buffer From Unclarified CHO Feed

pH	NaCl (nM)	Imidazole (nM)
8.5	500	10
8.5	500	30
7.5	500	10
7.5	111	10
8.5	111	30
8	305	20
7.5	111	30
8.5	111	10
8	305	20
7.5	500	30

## Results

### CHO Cell Passage Through Radial Flow Column

The average diameter of CHO cells (14.02–15.21 μm; Han et al., 2006) was used to select frit pore sizes for the column. Frits were sized so as to enable CHO cell passage without allowing the 300 μm IMAC beads to escape the column. Pores of 40 μm and 200 μm were chosen for pilot experiments using shake flask feed stocks of CHO‐S at a density of 1 × 10^6^ cells/mL (97.4% viable). Results from these experiments showed that the cell density of the feedstream passed through the 40 μm pore size frit dropped by 35% and the remaining cells had a reduced viability (19.38% ± 2.3%). By contrast, cells remained viable (97%± 2.2%) when the feed was passed through a 200 μm pore size frit. All further experiments were, therefore, carried out using the 200 μm pore frit.

**Figure 2:**
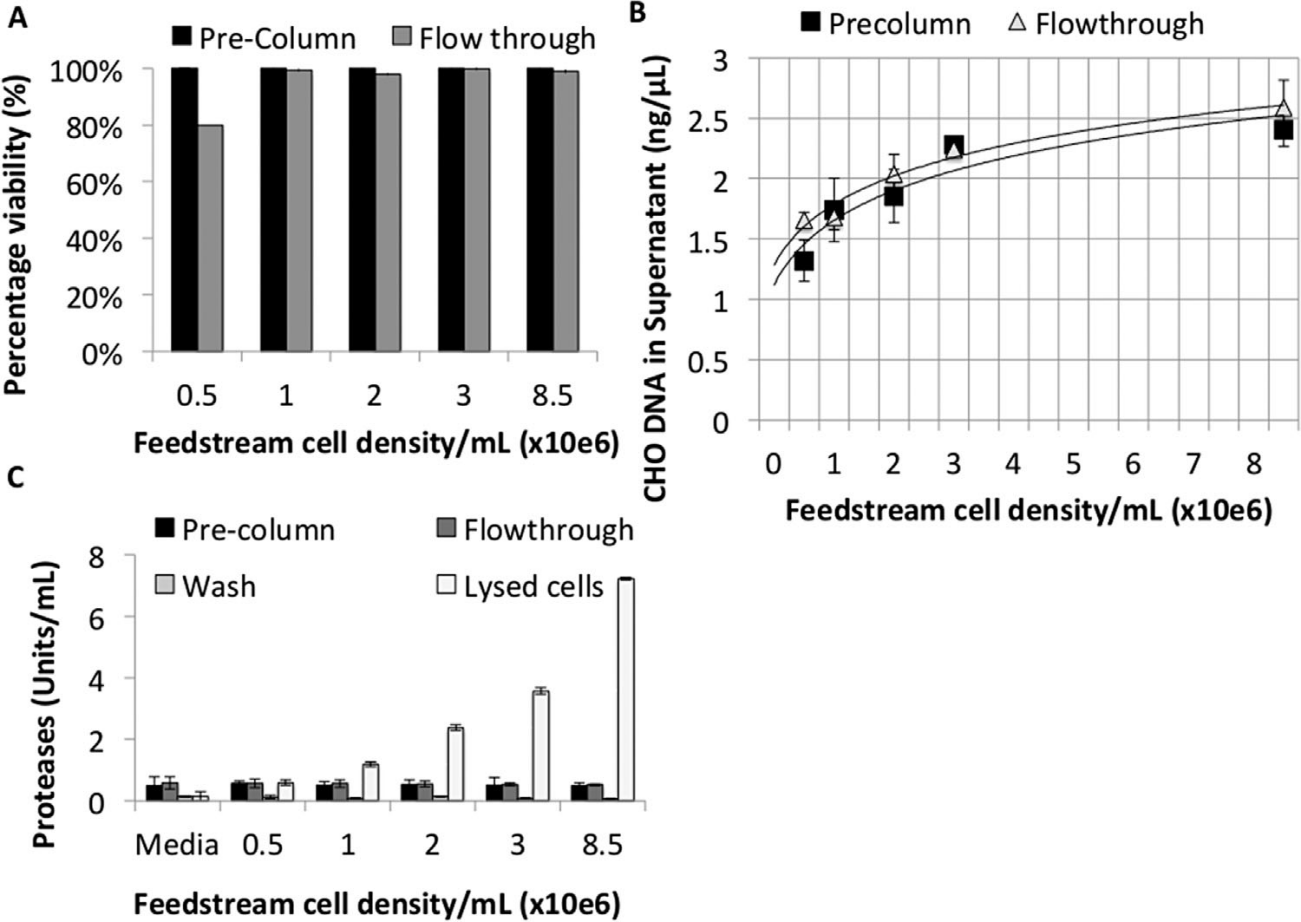
Analysis of unclarified feed streams containing different densities of CHO cells before and after passage through a scale down RFC with large bead resin (n = 3). **A**: Percentage cell viability of unclarified feed stream in pre‐column, flow through and washing conditions. **B**: qPCR quantification of host cell DNA in pre‐column and flow through conditions. **C**: Quantification of supernatant protease activity in unclarified feed stream and maximum cell lyses samples.

The effect of increased cell density on cell passage through the column was analyzed using shake‐flask feeds with 0.5–8.5 × 10^6^ cells/mL. Greater than 98% of cells remained viable in the flow‐through from feed streams containing >1 × 10^6^ cells/mL (Fig. 2A). This data was confirmed via analysis of cell lysis indicators including host cell DNA (hDNA) and proteolytic activity. The same markers showed no significant difference between pre/post column samples (*P *> 0.05) (Fig. 2B and C).

**Figure 3:**
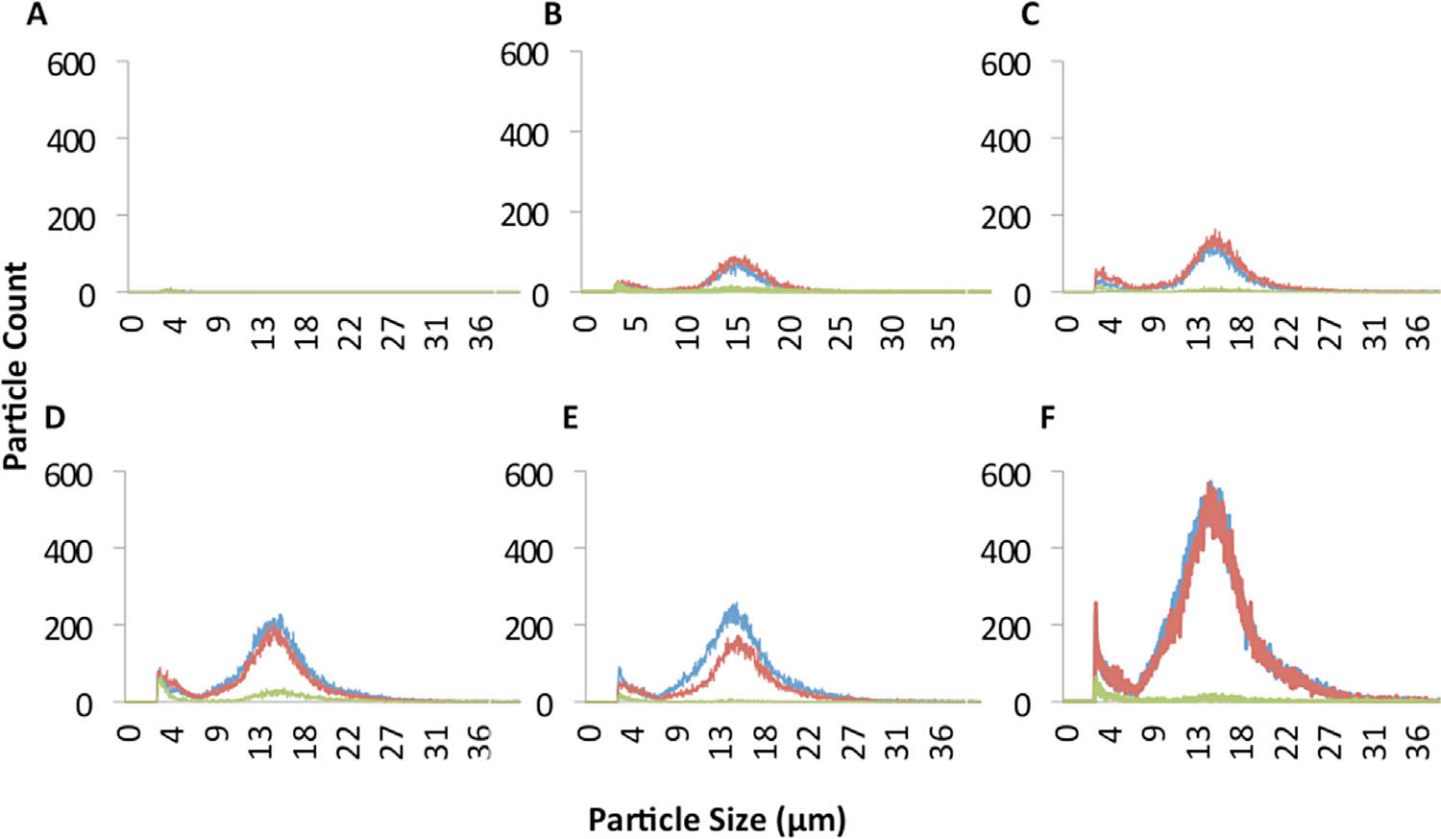
Particle size distribution of crude feed streams applied to a scale down radial‐flow column packed with large bead resin. Feed stream cell density; (**A**) media only, (**B**) 0.5 × 10^6^ cells/mL, (**C**) 1 × 10^6^ cells/mL, (**D**) 2 × 10^6^ cells/mL, (**E**) 3 × 10^6^ cells/mL, (**F**) 8.5 × 10^6^ cells/mL. Blue indicates pre‐column sample, red flow through, and green PBS wash.

Cell integrity was assayed via examination of the particle size distribution of feed streams pre and post column. Counts of particulate matter from cell lysis and protein aggregation (<6.7 μm) and larger particles such as cell aggregates (>22 μm) increased in proportion with cell density. Differences in the size distribution of samples from pre‐column and flow‐through samples were low in feed streams containing 0.5, 1, 2, or 8.5 × 10^6^ cells/mL, which is consistent with previous results. Washing of the column resulted in effective DNA clearance (zero detection via qPCR), a decrease in proteolytic activity to <2 U/mL and a low particulate count in all conditions (Fig. 2B, C and Fig. 3). These data verified that CHO cells survived passage through the column without significant detrimental effects.

### IMAC Capture of Model His‐Tagged Protein From Fermentation Media

**Figure 4:**
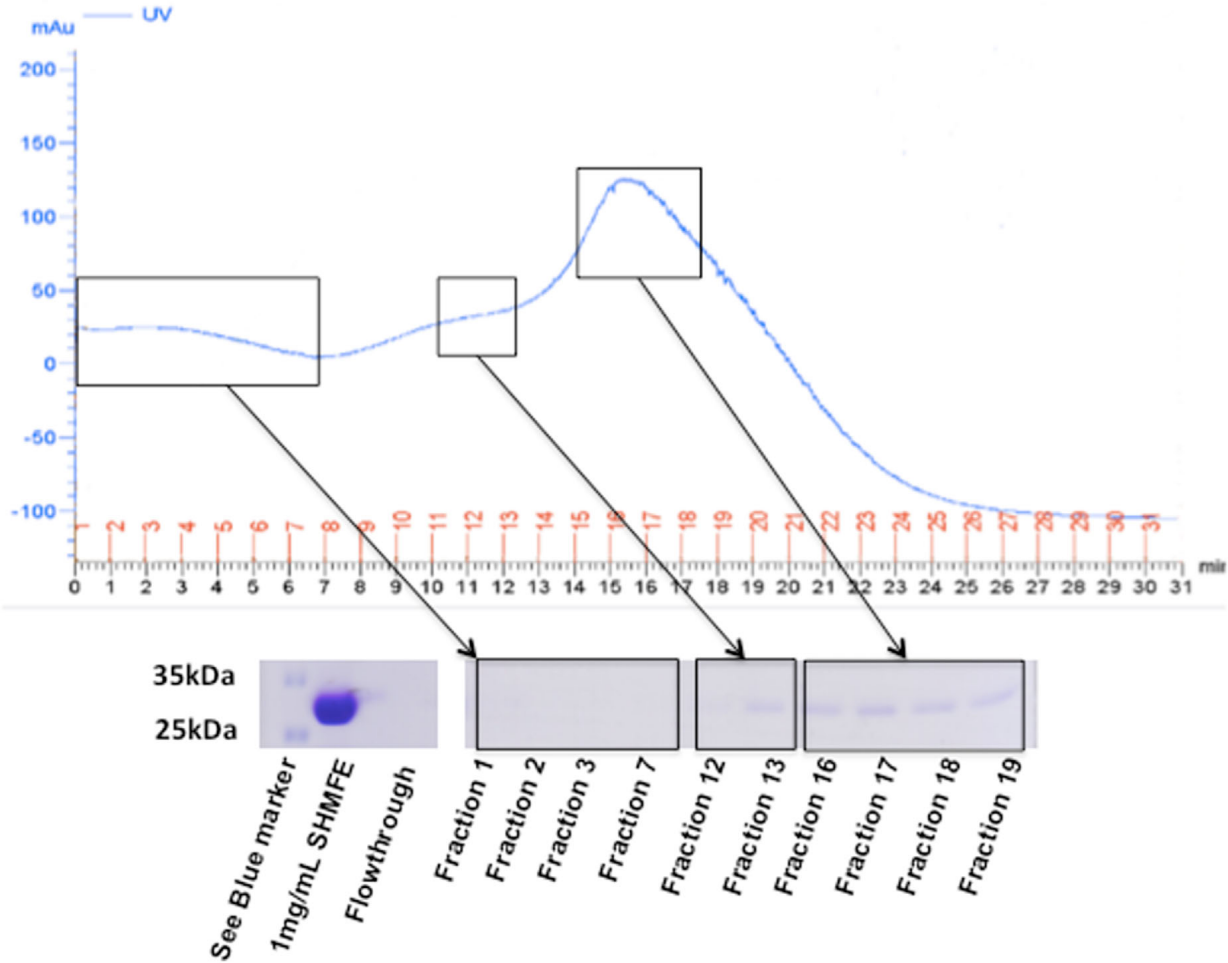
FPLC and SDS–PAGE separation of eluted fractions of a model His‐tagged single‐chain variable fragment from conditioned media buffered to IMAC binding conditions. Red numbers indicate 1 mL fraction collection.

Recovery of His‐tagged proteins is dependent on the composition of media from which it is purified; accordingly, media is usually buffered to favor IMAC binding conditions prior to purification. We simulated IMAC capture at the end of CHO‐S fermentation using media from a 12‐day‐old non‐transfected shake flask culture that had been clarified and spiked with 1 mg/mL His‐tagged ScFv (His‐SM3E). Protein recovery using the radial flow bed was analyzed using both un‐buffered and buffered media conditions. Un‐buffered media received no addition prior to chromatography whilst buffered media was adjusted to 150 mM NaCl, pH >7.5 with 20 mM imidazole. Total product recovery was improved in buffered media, 83% versus 61%, as determined by absorbance at 280 nm (data not shown). UV absorbance and SDS–PAGE resolution of protein captured from buffered media showed that the main protein fraction was eluted in CV3 and CV4. There was no visible protein band in the column flow‐through suggesting adequate binding to the IMAC matrix (Fig. 4). The model protein was successfully recovered from media conditioned to simulate typical harvest conditions, showing that His‐tag recovery was feasible using the combination of large bead resin and scaled down radial flow column.

### Buffer Optimization for His‐Tagged CEA Recovery

**Figure 5:**
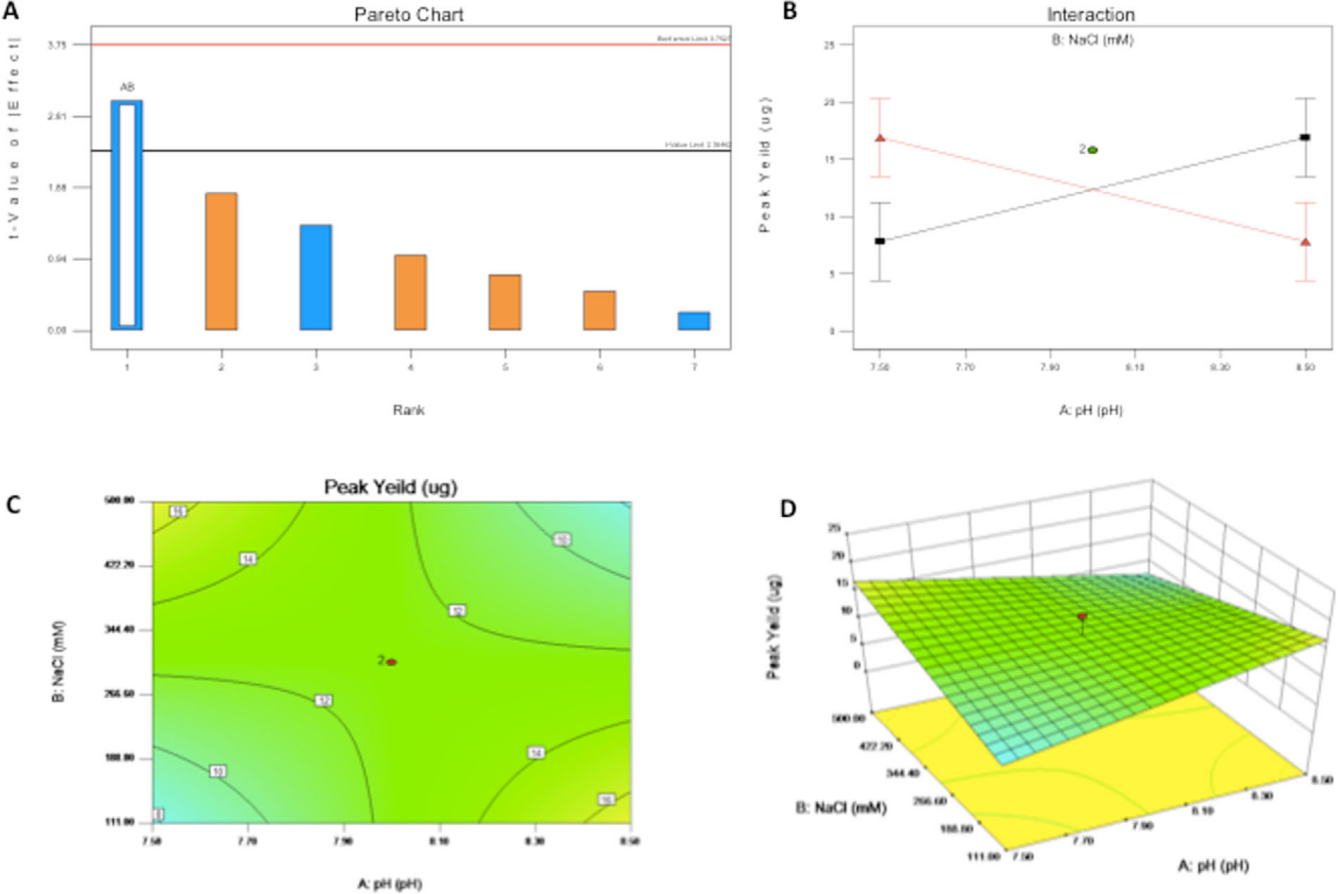
Design of experiments based optimization of buffer composition for IMAC capture of His‐tagged CEA. **A**: Pareto Chart indicates significant factor interactions above 1 value limit (black line). Blue indicates negative effects, orange positive effects and hollow significant effects (Factor labels; a = pH, b = NaCl, c = imidazole). **B**: Factor interaction of NaCl and pH (Red = 500 mM NaCl, Black = no NaCl addition). **C**: 2D, and (**D**) 3D surface response curve of His‐tagged CEA capture.

Having established that His‐tagged proteins could be recovered from buffered media, we focused on recovery of the target protein after expression by CHO‐CEA cells. Buffer optimization was performed in order to achieve maximum recovery of the target protein from clarified shake flask supernatant. DesignExpert8 (Shukla et al., 2001) was used to determine the necessary experimental parameters for optimizing buffer composition. A central composite, two‐level, three‐factorial design was used to investigate the effects of pH, NaCl, and imidazole concentration on product recovery. One significant factor interaction was found between pH and NaCl concentration (*P* < 0.05) (Fig. 5A and B). Percentage recovery ranged between ∼36–73%. Reducing NaCl concentration at high pH and increasing it at lower pH showed equally improved recovery of CEA (Fig. 5C and D). The lower pH of 7.5 was selected for further experiments to reduce cell stress whilst imidazole was maintained at a concentration of 20 mM after no effect was found on CEA yield. However purification at pH 7.5 meant that a high concentration of NaCl was required (500 mM) with potential negative effects on cell integrity.

### Cell Survival in Optimized IMAC Buffer Conditions

**Figure 6:**
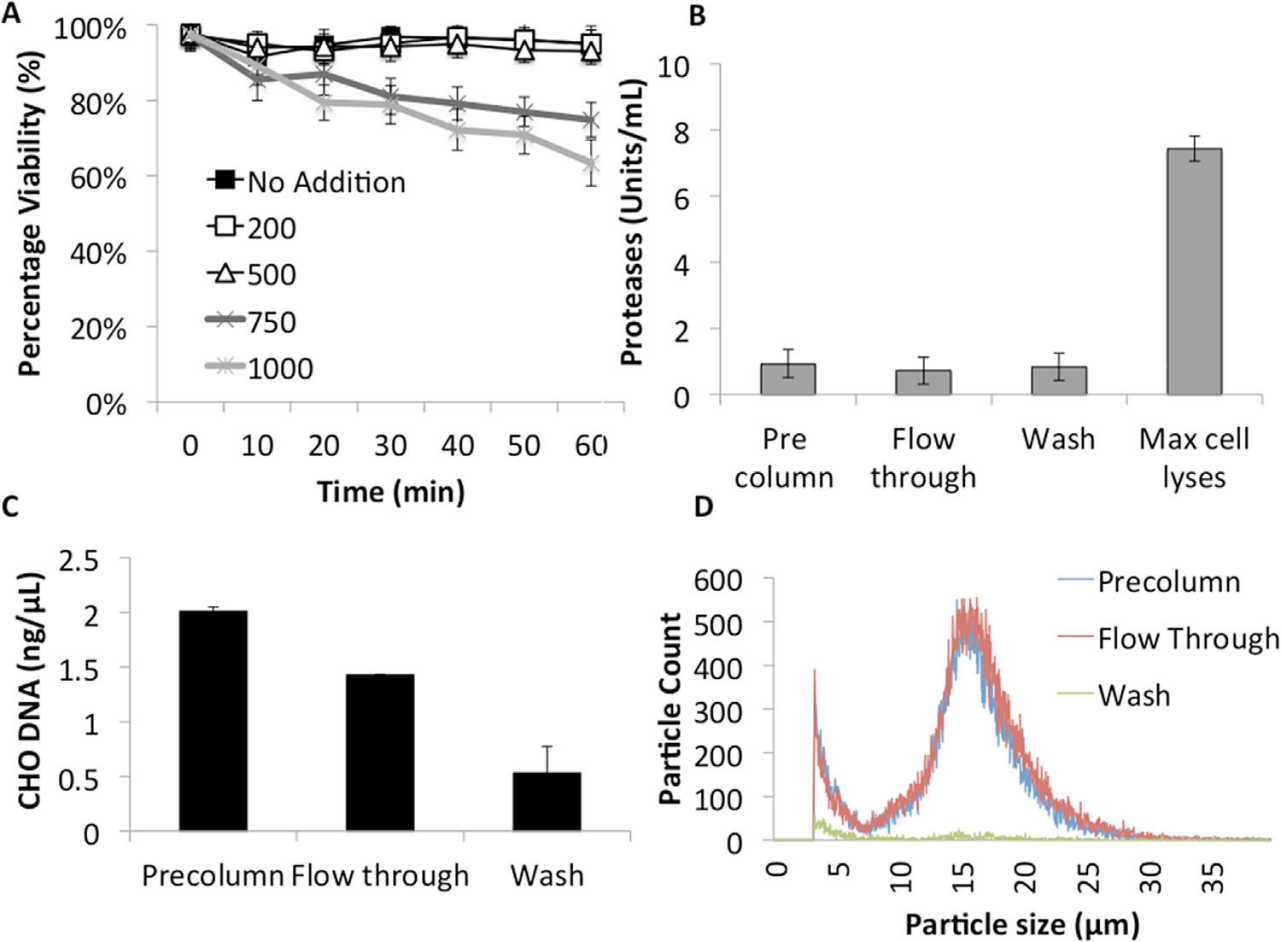
Cell survival and column flow characteristics of feed stream under optimized buffer conditions. **A**: CHO cell survival in buffered media with increasing NaCL concentration over 60 min. **B**: Protease activity in unclarified feed stream and maximum cell lyses samples after column flow buffered with 500 mM NaCl, 20 mM imidazole, and pH 8.5. **C**: Host cell DNA in pre‐column, flow through, and wash samples. **D**: PSD of feed stream from column buffered with 500 mM NaCl, 20 mM imidazole, and pH 8.5.

We next investigated the effects of high salt concentration and IMAC buffering on feed characteristics and cell survival. Viability was reduced by ∼2% and 5%, respectively, over 1 h in shake flasks with 200 mM and 500 mM NaCl, whilst feeds exposed to 750 mM and 1,000 mM NaCl reduced by as much as 34% (Fig. 6A). Consequently binding buffer composed of 500 mM NaCl, 20 mM imidazole, and a pH of 7.5 was selected for use in further experiments. CHO‐S cells in IMAC buffered media were passed through the column to determine the impact on feed characteristics. Percentage viability in the flow‐through remained >98% whilst host cell contaminants remained low (protease activity ∼8 U/mL and 2 ng/μL hDNA) (Fig. 6B and C). Particle size distribution corroborated these results showing no significant difference (*P* > 0.05) between pre‐column and flow‐through samples (Fig. 6D) as seen previously with passage of CHO‐S cells through the column. Our results indicated that the optimized IMAC buffer did not substantially affect cell characteristics, nor increase protease levels in the feed or eluted fractions.

### Comparing Recovery of His‐Tagged CEA From Unclarified and Clarified Shake Flask Feed Streams

**Figure 7:**
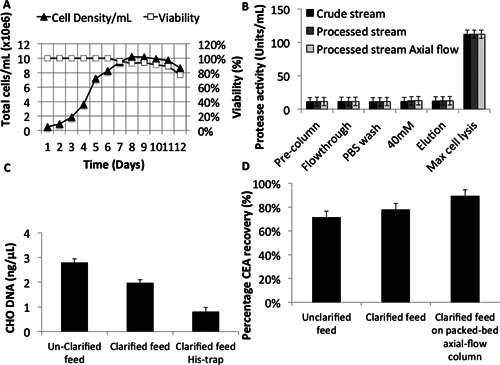
Comparison of crude and processed feed applied to a RFC system packed with large diameter resin. **A**: Fed‐batch shake flask growth of CHO cells expressing His‐tagged CEA. **B**: Viable cells/mL before and after passage through column. **C**: Protease activity in crude and processed feed streams. **D**: Percentage recovery of His‐tagged CEA from crude and processed feed streams.

Once the optimum buffer composition had been chosen, we investigated the effect of media clarification on His‐tagged CEA recovery in the radial flow column and compared this with recovery from a commercial axial flow column (HisTrap). CHO cells expressing His‐tagged CEA were cultivated in fed‐batch shake flask conditions for 12 days (Fig. 7A). Feed was buffered and applied to the column either clarified or unclarified. Cells from the unclarified feed remained ∼97% viable in column flow‐through with low contamination levels of ∼7.5 U/mL protease activity and >2 ng/μL hDNA (Fig. 7B and C) and a particle size distribution that was indistinguishable from results shown previously (Figs. 3 and 6D).

No difference in proteolytic activity was observed between unclarified and clarified feeds (Fig. 7B), however, the hDNA quantity decreased in both clarified conditions, (1.95 ng/μL from radial flow and 0.78 ng/μL from HisTrap columns compared to 2.77 ng/μL from unclarified feed). Protein recovery was higher from clarified media than from unclarified media (Fig. 7C), with the standard packed‐bed HisTrap column achieving ∼89% recovery, the radial flow ∼78% and unclarified feed ∼71% (Fig. 7D).

### Integrated His‐Tagged CEA Recovery From Bioreactor Fermentation

**Figure 8:**
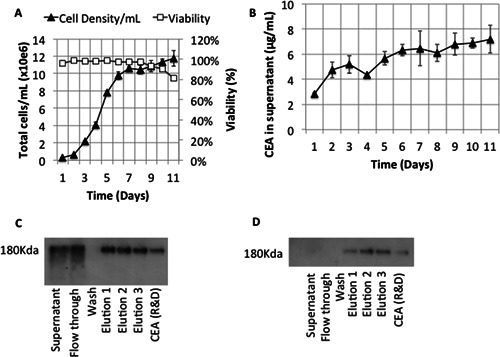
Primary capture of His‐tagged CEA from 3 L fed‐batch bioreactor fermentation. **A**: Fed‐batch fermentation of CHO in SUT bioreactor. **B**: Time course of CEA expression in the bioreactor. **C**: Anti‐CEA Western blot of column eluate. **D**: Anti‐His Western blot of column eluate. All elutions and positive control were diluted to 1 μg/mL prior to western blotting.

Integration of the column and bioreactor was carried out using fed‐batch fermentation. The bioreactor feed had an increased cell density and product titer compared to shake flask growth (11.8 × 10^6^ cells/mL and 7.2 μg/mL CEA) (Fig. 8A and B). Feed was harvested via passage directly through the column in 1 L batches with subsequent regeneration to ensure consistency (see Methods Section). Cell viability and feed stream characteristics were not significantly different to previous experiments. Average product recovery was 69% (±6%) in repeated batch purifications giving a final pooled sample yield of 14.6 mg as determined by ELISA. Fractions from individual purifications were diluted to 1 μg/mL for Western blotting and were visualized using both anti‐CEA and anti‐His antibodies (Fig. 8C and D). There was no visible difference between subsequent purifications. Bands for CEA, but not His‐tag, were identified in flow‐through from the column indicating that His‐tag recovery was efficient and the apparent lower yield compared to equivalent shake flask material was due to untagged or degraded CEA passing through the column.

## Discussion

Primary capture of recombinant proteins from mammalian cells is a lengthy procedure with potential for gains in efficiency, spurred by the increasing focus on manufacturing (Farid, 2007), which is currently estimated at 50–80% of total process costs (Levy et al., 2014). We have addressed this by developing an integrated primary capture step for recovery of secreted, His‐tagged recombinant proteins from unclarified mammalian cell feeds using RFC operated with large diameter resins. As yield is a function of the number of processes (Farid, 2011), it is advantageous to remove process steps where possible. Process efficiency can be achieved by improved integration of steps immediately following fermentation (Warikoo et al., 2012), with the added benefit of reducing process volumes, CAPEX, operator costs, and consumables.

In this study IMAC purification was utilized for the recovery of a complex glycoprotein from CHO cells. Demand for CEA has been met previously by recovery from ascetic fluids (Hammarström, 1999, Sigma–Aldrich). Attempts to express it in the yeast *P*. *pastoris* have achieved expression of only truncated forms of the protein (Hellwig et al., 1999; Sainz‐Pastor et al., 2006; Tolner et al., 2013). Consequently CEA, like many other recombinant proteins, requires expression in mammalian or glyco‐engineered insect cells to ensure correct glycosylation for structure and function. CEA is currently available commercially from recombinant or human sources and is widely used in diagnostic applications; however, it remains an expensive protein with inconsistent glycosylation profiles. As with other proteins that lack a natural purification tag the application of a His fusion tag is a simple and convenient purification technique.

During this study, a number of feed stream factors were taken into account that could affect protein yield or quality. Cell damage plays an important role in the characteristics of feed streams (Anspach et al., 1999; Salte et al., 2006) and has been shown to vary widely across bioprocess operations (Hogwood et al., 2013; Kim et al., 2013). We focused specifically on proteolytic activity and hDNA contamination rather than host cell protein (HCP) content as this is not directly linked to product degradation (Cordoba et al., 2005). By contrast, proteases directly affect product stability and affinity tag cleavage (Gao et al., 2011). Contamination with hDNA significantly affects feed viscosity (Balasundaram et al., 2009), and therefore, column pressure drop as well as needing to meet stringent regulatory standards in final preparations (Nissom, 2007). In our process, cell damage was predicted to occur during column passage, especially as the cells traversed the relatively high shear regions of the frit. Our results showed a loss of viability of between 2% and 4% and no significant difference between particle size distribution, protease activity, or hDNA content in pre and post column samples. By comparison, previous results of direct capture using expanded bed adsorption (EBA) from hybridoma cells showed an increase in hDNA contamination of up to 54% (Feuser et al., 1999).

The benchmark for primary protein recovery is currently axial packed‐bed chromatography; our results demonstrated that percentage recovery of CEA was 17.9% greater in an axial packed‐bed column (89.16%) compared to capture from unclarified feed using the large diameter resin in radial flow format (71.6%). In industrial processes, His‐tag recovery can be as high as 90% (Saraswat et al., 2013). In this study, three buffer factors (pH, imidazole, and NaCl concentration) were optimized for product capture, however, a broader optimization of process factors (bead diameter, bed height, load mass, flow rate, cell density, temperature etc.) would be expected to improve yield.

Our process utilized IDA chelating agarose beads of between 300 μm and 500 μm in diameter forming a packed resin charged with nickel. However, a range of resins are available differing in construction, functional groups, bead size, and packing density (Bornhorst and Falke, 2000; Hochulial, 1987; Saraswat et al., 2013; Warren and Bettadapura, 2005). These variables are expected to have a significant impact on performance and feed stream characteristics, for example, bead diameter will directly affect antiparticle porosity affecting cell travel through the column, fluid dynamics, and the shear rate experienced by the cells thus affecting protein yield.

The improvement in process efficiency yielded from using an integrated capture step would result in a reduction in COG/g and has been shown previously with capture techniques from unclarified feeds (Schügerl and Hubbuch, 2005). EBA, a format with similar process advantages to large‐bead resins, has been compared with standard bioprocess techniques resulting in reduced COG/g and simplified recovery in microbial and mammalian cell downstream processing (Chhatre et al., 2006; Tolner et al., 2013; Willoughby et al., 2004). Similar efficiency gains from the removal of clarification steps could be expected with large bead diameter resins. Uptake of novel techniques can be affected by concerns over regulatory compliance however the combination of RFC and EBA has been applied to unclarified microbial fermentation broths using His‐tagged proteins previously in cGMP‐compliant processes (Tolner et al., 2013), showing that adoption of similar approaches does not necessarily introduce regulatory hurdles.

## Conclusions

In conclusion, primary recovery of recombinant proteins from unclarified media is an attractive processing technique with the potential to significantly improve process efficiency. This study demonstrates primary capture of heavily glycosylated, His‐tagged CEA using large bead radial flow Ni‐IMAC chromatography without detrimental affects on cell viability. Our approach could be applied to the recovery of many different proteins from mammalian feed streams using other modes of chromatographic interactions such as affinity and ion exchange chromatography.

This study was supported by the Debbie Fund and the UCL Cancer Institute Research Trust, the KCL and UCL Comprehensive Cancer Imaging Centre (CCIC) funded by Cancer Research UK (CRUK) and the Engineering and Physical Sciences Research Council (EPSRC) in association with the Medical Research Council (MRC) and Department of Health (DoH) (England), the DoH and CR‐UK Experimental Cancer Medicine Centre (ECMC), EU Seventh Framework Programme (FP7), the DARTRIX project and IMAGINT and the National Institute for Health Research University College London Hospitals Biomedical Research Centre (NIHR BRC).

The funders had no role in study design, data collection and analysis, decision to publish, or preparation of the manuscript.
